# The Chemical Profiling and Immunological Activity of Polysaccharides from the Rhizome of *Imperata cylindrica* Using Hot Water Extraction

**DOI:** 10.3390/molecules30122635

**Published:** 2025-06-18

**Authors:** Meng-Ge Sun, Jia-Jie Chen, Jia-Min Xu, Wei Chen, Xiao-Bing Chen, Dong-Sheng Yang

**Affiliations:** 1 College of Life Science, Zhuhai College of Science and Technology, Zhuhai 519041, China; sunmg22@mails.jlu.edu.cn (M.-G.S.); if701429asd@163.com (J.-J.C.); a985251037@163.com (J.-M.X.); smuchenwei@foxmail.com (W.C.); 2 College of Life Science, Jilin University, Changchun 130012, China

**Keywords:** rhizome of *Imperata cylindrica*, polysaccharides, structural characterization, RAW264.7 cells, immunological activity

## Abstract

To investigate the immunomodulatory activity of polysaccharides derived from the rhizome of *Imperata cylindrica*, polysaccharides (IRPs-H) were extracted using hot water extraction and further purified via DEAE-52 ion-exchange chromatography, yielding three fractions: IRPs-H1, IRPs-H2, and IRPs-H3. The structural features of these fractions were characterized by Fourier-transform infrared spectroscopy (FT-IR), high-performance gel permeation chromatography (HPGPC), atomic force microscopy (AFM), and thermogravimetric analysis (TGA). Their immunological activities were evaluated in vitro. All three fractions were identified as neutral pyranose-type polysaccharides, primarily composed of glucose and xylose, exhibiting good thermal stability and lacking long-chain structures. In vitro assays using RAW264.7 macrophages demonstrated that these polysaccharides promoted cell proliferation (50–800 μg/mL), enhanced phagocytic activity, and induced morphological changes characteristic of macrophage activation, including irregular shapes and pseudopod formation. ELISA and flow cytometry analyses revealed dose-dependent increases in nitric oxide (NO), interleukin-6 (IL-6), tumor necrosis factor-α (TNF-α), and reactive oxygen species (ROS) levels. Notably, the IRPs-H3 fraction stimulated TNF-α and IL-6 production to levels of 438.02 ± 14.14 pg/mL and 30.13 ± 1.27 pg/mL, respectively, which were comparable to those induced by lipopolysaccharide (LPS), the positive control (460.83 ± 16.10 pg/mL and 31.87 ± 1.72 pg/mL, respectively). These results suggest that polysaccharides extracted from the rhizome of *Imperata cylindrica* possess significant immunostimulatory properties and hold potential for development as functional food ingredients or immune-enhancing agents.

## 1. Introduction

Polysaccharides are high-molecular-weight carbohydrates consisting of multiple monosaccharide units linked via glycosidic bonds. They are ubiquitously distributed in nature, with plant-derived polysaccharides representing a major source of food-grade polysaccharides [1]. Owing to their wide availability, low production cost, and favorable safety profile, polysaccharides have attracted considerable scientific interest. They are well recognized for their diverse pharmacological activities, including antioxidant, antitumor, and anti-inflammatory effects, making them highly valuable for applications in the food, pharmaceutical, and biomedical industries [2].

Studies have demonstrated that plant polysaccharides regulate immune function through multiple mechanisms, primarily by activating macrophages [3]. Their immunomodulatory effects include promoting macrophage proliferation, differentiation, and phagocytosis. This is achieved by binding to pattern recognition receptors (PRRs) on the cell membrane, such as Toll-like receptors (TLRs) and C-type lectin receptors (CLRs), which activate downstream signaling pathways [4,5]. Consequently, macrophages secrete immune mediators like NO, TNF-α, and IL-6. These molecules enhance innate immunity and modulate adaptive immune responses, improving defense against pathogens and tumor cells. Moreover, plant polysaccharides stimulate cytokine and chemokine secretion from immune cells, activating T and B lymphocytes and strengthening antigen-specific immunity [6]. They also regulate the balance of T cell subsets, particularly the Th1/Th2 ratio, further boosting immune responses. By inducing a dominant Th1 response, polysaccharides exhibit antitumor activity [7]. Additionally, they contribute to immune tolerance by preventing autoimmune attacks and reducing damage caused by excessive immune activation, thus helping to suppress autoimmune diseases. Overall, plant polysaccharides possess significant potential for immune regulation, cancer therapy, and immune tolerance modulation.

With the increasing emphasis on health and wellness, research on medicinal and edible plants has garnered widespread attention. To date, investigations have primarily focused on small-molecule bioactives such as flavonoids and polyphenols, owing to their well-documented pharmacological properties. In recent years, however, polysaccharides have emerged as a promising new frontier in the study of these dual-use botanicals, driven by their diverse pharmacological activities and potential health benefits. Significant progress has been made in elucidating the structure–activity relationships and biological functions of plant polysaccharides, particularly in the realms of immunomodulation, antioxidation, anti-inflammation, and antitumor activity.

The rhizome of *Imperata cylindrica* Beauv. var. *major* (Nees) C.E. Hubb., a member of the Poaceae family, is widely distributed across China, particularly in the northeast, north, east, central, and southwestern regions [8]. The rhizome of *Imperata cylindrica* is a widely used traditional Chinese medicinal herb with abundant wild resources and high nutritional and therapeutic value. Traditionally, its rhizomes are harvested from the wild, cleaned, chopped, and processed by juicing or boiling. The *Imperata cylindrica* rhizome is traditionally valued for its blood-cooling, hemostatic, and diuretic properties. It is commonly used to treat hematuria, nephritis, and urinary tract infections associated with heat toxins. Its medicinal uses are documented in classical texts such as *Mingyi Bielu* and *Tujing Bencao*. Recent pharmacological studies have identified key bioactive compounds, including polysaccharides, flavonoids, triterpenoids, sterols, organic acids, anthraquinones, and lactones [9]. These constituents contribute to the rhizome’s antioxidant, antibacterial, anti-inflammatory, antitumor, and hepatoprotective effects [10,11,12]. Currently, the *Imperata cylindrica* rhizome is widely used as both a traditional medicine and food source, often incorporated with other herbs into products such as beverages, pharmaceuticals, and supplements.

Although the *Imperata cylindrica* rhizome has a long history of use, extant research remains largely confined to crude extracts, which limits its full development and utilization [13]. Among the various bioactive constituents, polysaccharides constitute over 80% of the total extractable material, yet studies on these macromolecules are scarce and largely restricted to extraction protocols. Reports on their isolation, purification, and structural characterization are limited, and systematic investigations into their biological activities are lacking. While preliminary studies have demonstrated that *Imperata cylindrica* rhizome polysaccharides exhibit antioxidative, antibacterial, anti-inflammatory, antitumor, hepatoprotective, and renoprotective effects, comprehensive evaluations of their immunomodulatory potential have not yet been reported. In this study, we isolated and purified three IRPs-H fractions from the rhizome of *Imperata cylindrica*. We characterized their monosaccharide composition and structural features using FT-IR, AFM, and TGA. We then evaluated their immunological effects on RAW264.7 macrophages, assessing proliferation, differentiation, phagocytosis, morphological changes, NO, TNF-α, IL-6, and ROS levels. Our results elucidate the cellular and molecular mechanisms by which *Imperata cylindrica* polysaccharides activate macrophages.

## 2. Results and Discussion

### 2.1. The Separation and Purification Results of IRPs-H

In this study, polysaccharides were extracted from *Imperata cylindrica* rhizomes using a hot water extraction method, yielding a recovery rate of 5.16%. IRPs-H was fractionated using a DEAE-52 cellulose column with a stepwise elution of distilled water, 0.2, and 0.4 mol/L NaCl, yielding IRPs-H1, IRPs-H2, and IRPs-H3, respectively. No absorbance peak was observed with 0.6 mol/L NaCl. Therefore, IRPs-H1, IRPs-H2, and IRPs-H3 were selected for further analysis.

### 2.2. Structural Analysis of Different IRPs-H Polysaccharides

#### 2.2.1. Analysis of UV Spectroscopy

**Figure 1:**
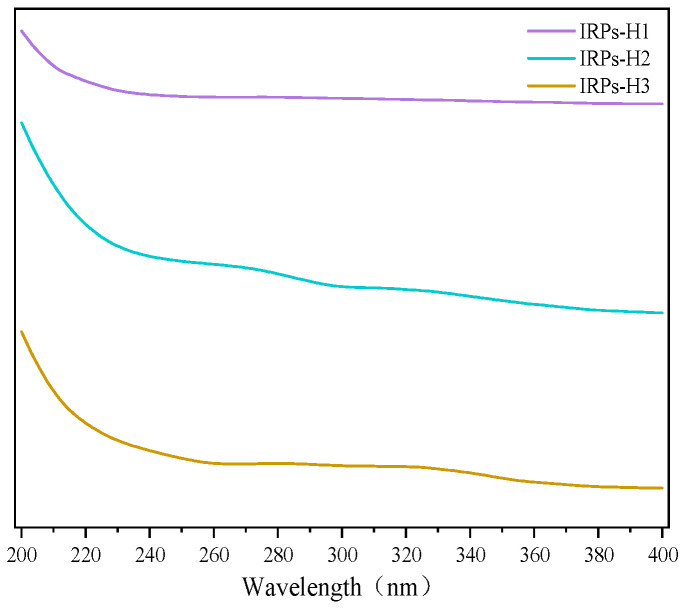
The results of ultraviolet spectra of IRPs-H.

UV spectrophotometry is a widely used method for assessing the purity of polysaccharides. Nucleic acids and proteins exhibit characteristic absorbance at 260 nm and 280 nm, respectively, due to the presence of conjugated double bonds in nucleotides and aromatic amino acids such as tryptophan and tyrosine. Polysaccharides, which lack these chromophores, typically show no significant absorbance at these wavelengths. Therefore, the absence of absorption peaks at 260 and 280 nm in UV spectra indicates that the polysaccharide samples are free from nucleic acid and protein contamination [14]. The UV absorption spectra of the IRPs-H components in the wavelength range of 200~400 nm are shown in Figure 1. The results showed that IRPs-H1, IRPs-H2, and IRPs-H3 had no obvious absorption peaks at 260 and 280 nm, which proved that the three polysaccharides did not contain nucleic acids and proteins.

#### 2.2.2. Analysis of FT-IR Spectroscopy

**Figure 2:**
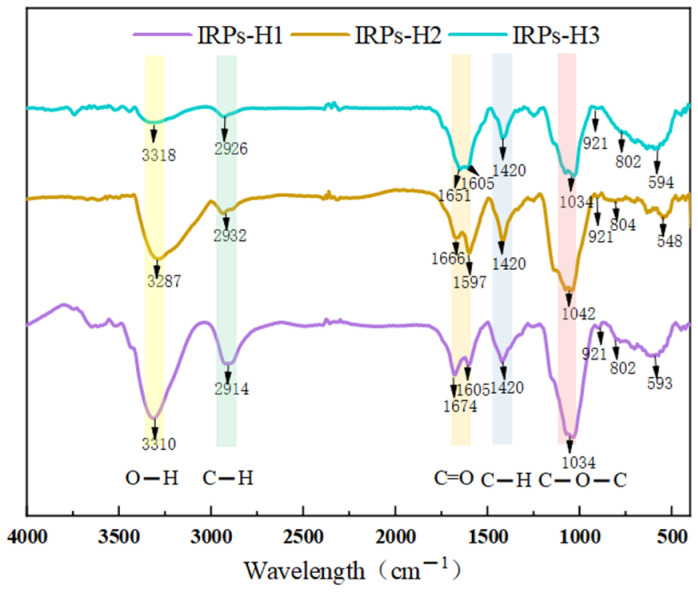
FT-IR spectra of different IRPs-H polysaccharides.

Figure 2 shows the infrared spectra of IRPs-H1, IRPs-H2, and IRPs-H3, displaying characteristic polysaccharide absorption bands in the regions of 3600–3200 cm^−1^, 3000–2800 cm^−1^, 1500–1200 cm^−1^, and 1200–800 cm^−1^. The broad peaks at 3310 cm^−1^, 3287 cm^−1^, and 3318 cm^−1^ correspond to O–H stretching vibrations, indicating abundant hydroxyl groups. Peaks near 2914 cm^−1^, 2932 cm^−1^, and 2926 cm^−1^ are assigned to C–H stretching vibrations. Absorption bands around 1666 cm^−1^, 1651 cm^−1^, and 1674 cm^−1^ represent C=O symmetric stretching of sugar ketones or water-bound polysaccharides. The peak at approximately 1420 cm^−1^ corresponds to symmetric COO^−^ stretching. Peaks at 1034 cm^−1^, 1042 cm^−1^, and 1037 cm^−1^ are characteristic of pyranose glycosides. The peak at 921 cm^−1^ indicates asymmetric ring stretching vibrations of the pyranose ring, suggesting β-glycosidic linkages, while the weak peak at 802 cm^−1^ is associated with α-glycosidic bonds. Finally, the band near 590 cm^−1^ arises from out-of-plane bending of the –OH group. Overall, the three fractions share similar structural features, including O–H, C–H, C=O groups, and sugar ring characteristic peaks [15]. Both α- and β-glycosidic bonds are present, consistent with a pyranose-type polysaccharide structure.

#### 2.2.3. Analysis of Monosaccharide Composition

The monosaccharide components of IRPs-H1, IRPs-H2, and IRPs-H3 were analyzed by triple quadrupole liquid chromatography-mass spectrometry (LC-MS). The results are shown in Table 1; all three components, IRPs-H1, IRPs-H2, and IRPs-H3, were identified as neutral polysaccharides, although their monosaccharide compositions varied. IRPs-H1 consists of Man, Rha, GalA, Glc, Gal, Xyl, and Fuc, with a molar ratio of 3.18:1.42:1.47:46.43:21.29:25.41:0.57. IRPs-H2 is composed of Man, Rha, Glc, Gal, Xyl, Rib, and Fuc, with a molar ratio of 1.61:3.74:34.75:5.80:42.88:6.54:4.53. IRPs-H3 contains Man, Rha, GlcA, Glc, Gal, Xyl, Rib, and Fuc, with a molar ratio of 0.47:16.85:3.11:48.01:4.05:17.04:2.33:8.13.

There are differences in both the proportion and types of monosaccharides present. IRPs-H1 is primarily composed of Glc, Gal, and Xyl; IRPs-H2 mainly consists of Glc and Xyl; and IRPs-H3 is mainly composed of Glc, Xyl, and Rha. Additionally, trace amounts of other monosaccharides such as GlcA, GalA, Rha, Rib, and Man are present in varying quantities. The uronic acid content in all three fractions is relatively low, confirming their classification as neutral polysaccharides. Investigating the relationship between monosaccharide composition and biological activity offers valuable insights for understanding polysaccharide structure–function correlations and guiding future structural characterization [16].

#### 2.2.4. Determination of Molecular Weight

**Figure 3:**
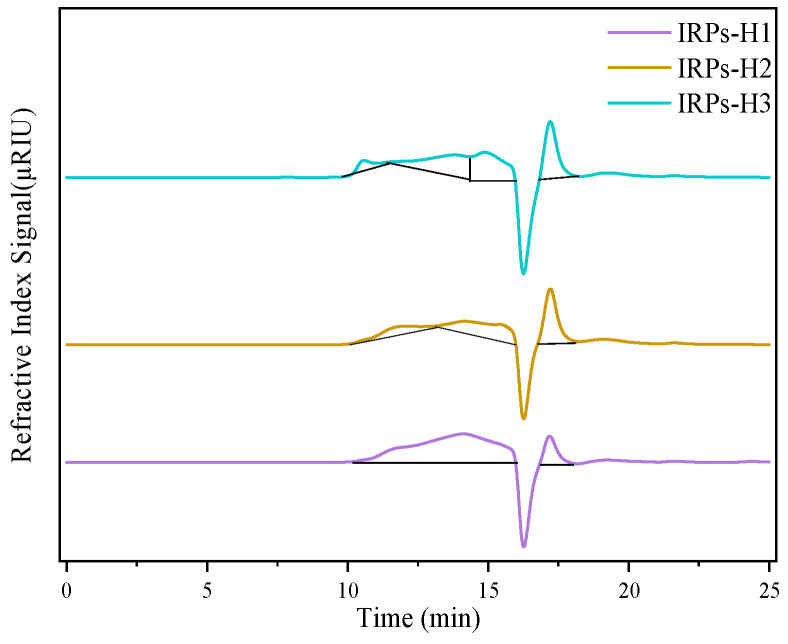
HPGPC with molecular weight distribution of different IRPs-H polysaccharides.

Using log (M/w) as the abscissa and the retention time as the ordinate, the linear correlation standard molecular weight linear equation of dextran was established as y = −2.3944x + 23.289, R2 = 0.9930 (linear equation molecular weight range 180–300,600 Da), and the molecular weight of polysaccharide was calculated according to the standard curve. According to the results of HPGPC analysis, combined with the analysis of Figure 3 and Table 2, the molecular weight range of polysaccharides in IRPs-H1, IRPs-H2, and IRPs-H3 samples showed significant differences, which was a polysaccharide with uneven molecular weight.

The molecular weight profile of IRPs-H1 is relatively simple, with two primary components at 6.9 kDa and 0.3 kDa, indicating a narrow distribution concentrated in the low to medium molecular weight range. In contrast, IRPs-H2 exhibits a more complex composition, with peaks at 66.55 kDa, 6.8 kDa, and 0.3 kDa, reflecting a broader distribution from high to low molecular weights. IRPs-H3 shows the most diverse molecular weight distribution, including one very high molecular weight fraction at 210.7 kDa and three additional fractions at 9.2 kDa, 3.3 kDa, and 0.3 kDa. This extensive range highlights the structural complexity of IRPs-H3. These distinct molecular weight characteristics may influence the functional and biological properties of each polysaccharide fraction, supporting their potential application in biomedical, pharmaceutical, and nutraceutical research.

While the molecular weight estimations were based on a well-established calibration curve using dextran standards, these standards do not fully replicate the branching patterns and conformational variability of natural polysaccharides. Therefore, the calculated molecular weights should be regarded as relative values. Additionally, due to limited sample availability, replicate injections and advanced verification techniques such as multi-angle light scattering (MALS) were not feasible in the current study. Despite these limitations, the retention time reproducibility and clear inter-sample differences support the overall reliability of the HPGPC results presented here.

#### 2.2.5. Analysis of AFM

**Figure 4:**
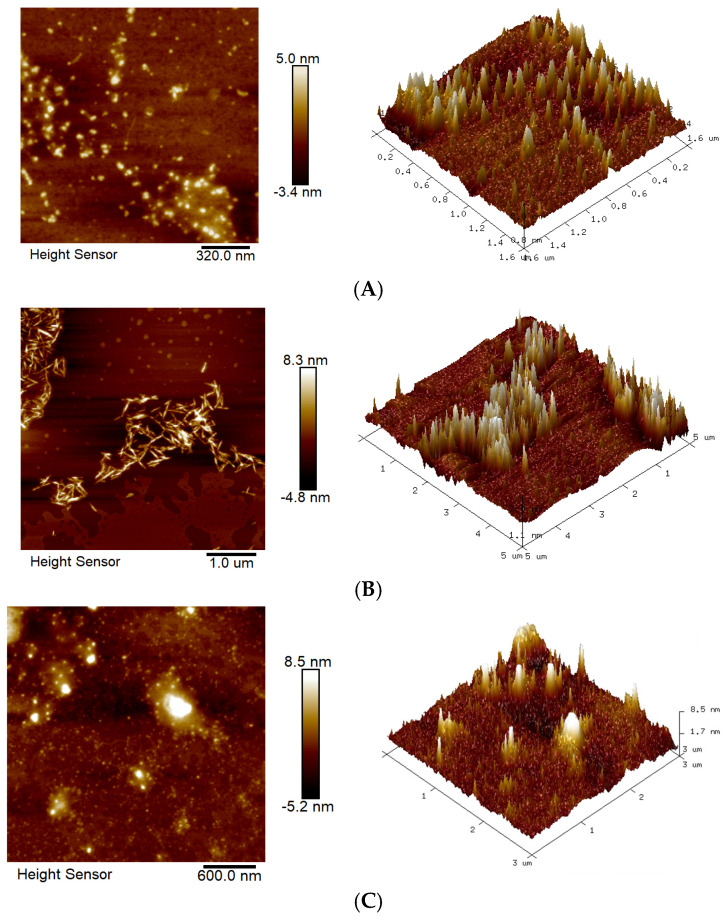
AFM images of different IRPs-H polysaccharides. (**A**) IRPs-H1; (**B**) IRPs-H2; (**C**) IRPs-H3.

The microscopic morphology of different IRPs-H polysaccharides in the solution was observed by AFM. Figure 4 shows two-dimensional and three-dimensional topography images of such components.

IRPs-H1 exhibited a relatively dispersed morphology, characterized by granular or agglomerated surface structures. The height variation between particles was approximately 5.0 nm, with an average spacing of around 320.0 nm, indicating a loosely distributed polysaccharide network. IRPs-H2 displayed a more ordered, elongated chain or fibrous structure, with height variations reaching 8.3 nm. The fibers extended up to 1.0 μm in length and width, suggesting a higher degree of molecular alignment and potential crosslinking. In contrast, IRPs-H3 showed a denser and more compact surface morphology, with tightly packed particles and a height variation of up to 8.5 nm. The average particle spacing was about 600.0 nm, indicating a more compact distribution likely influenced by van der Waals forces or other intermolecular interactions.

The 3D topographic images clearly reveal that all components exhibit a needle-like structure, forming irregular aggregates in aqueous solution. The surface of the samples displays significant undulations in certain regions, suggesting the presence of strong intermolecular interactions that may promote the crosslinking of monosaccharide molecules into aggregated structures [17].

#### 2.2.6. Analysis of Thermal Stability

**Figure 5:**
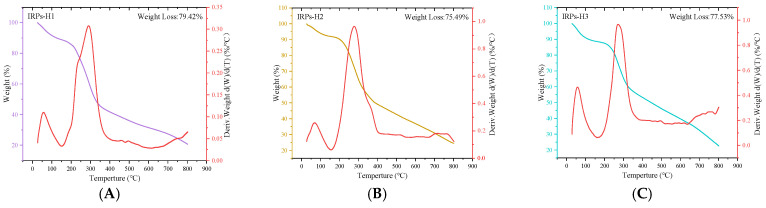
The TG-DTG curves of different IRPs-H polysaccharides were plotted. (**A**) IRPs-H1; (**B**) IRPs-H2; (**C**) IRPs-H3.

The correlation between mass change and temperature or time obtained through TGA provides valuable information on the thermal stability and compositional characteristics of polysaccharides [18]. Figure 5 presents the TGA results of the IRPs-H polysaccharide components. All three samples exhibited similar thermal decomposition profiles, with total mass losses ranging from 75% to 80%. The TGA curves revealed a multi-step decomposition process that can be divided into three distinct stages.

The first stage: the initial mass loss occurs in the temperature range of 30 °C to 150 °C, mainly due to the evaporation of free water and bound water in the sample.

The second stage: a large amount of mass loss occurs between 200 °C and 550 °C, which may be the result of the degradation of the chemical structure of polysaccharides. The serious destruction of the structure at this stage led to a sharp decline in quality.

The third stage: this involves gradual thermal degradation, primarily attributed to the breakdown of organic carbon components in the polysaccharides. As the temperature increases, the rate of mass loss decreases. Among the three samples, IRPs-H1 exhibited the highest total mass loss (79.42%), while IRPs-H2 showed the lowest (75.49%). Ultimately, the residual mass of each component stabilized at approximately 20% of the initial mass.

#### 2.2.7. Analysis of Triple Helix Structure

**Figure 6:**
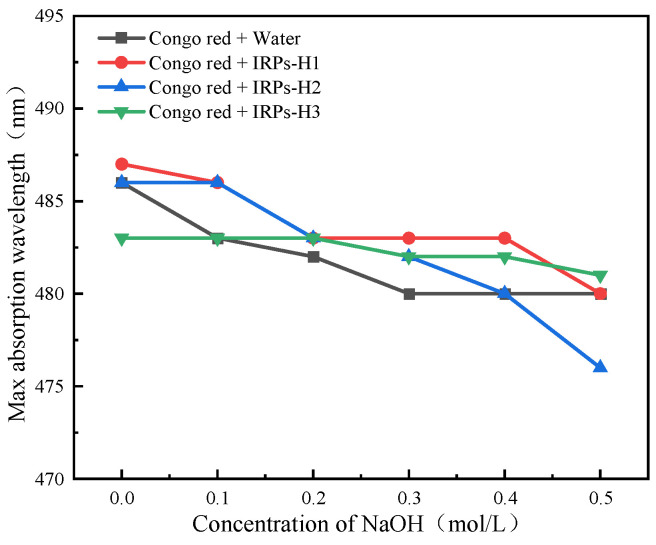
The maximum absorption wavelength changes in Congo red and three IRPs-H mixtures at different NaOH concentrations.

The Congo red assay is commonly used to evaluate the higher-order conformation of polysaccharides, particularly to detect triple-helix structures through a red shift in maximum absorption under alkaline conditions [19]. As shown in Figure 6, increasing NaOH concentrations led to a gradual decrease in the maximum absorption wavelengths of the Congo red–polysaccharide complexes across all IRPs-H components. No significant red shift (λmax) was observed compared to the blank control. These results indicate that IRPs-H1, IRPs-H2, and IRPs-H3 do not adopt a triple-helix conformation but are likely to exist in an irregularly coiled or disordered structure.

#### 2.2.8. Analysis of Branched Chain Structure

**Figure 7:**
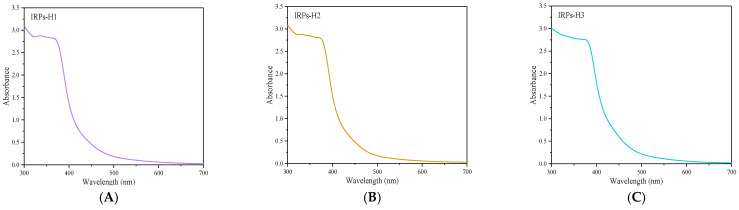
Scanning spectra of the reaction of IRPs-H polysaccharides with iodine-potassium iodide. Note: (**A**) IRPs-H1; (**B**) IRPs-H2; (**C**) IRPs-H3.

The UV spectra of the IRPs-H components after a reaction with an iodine reagent are shown in Figure 7. None of the polysaccharide–iodine complexes exhibited a characteristic absorption peak at 350 nm or 565 nm. This suggests the absence of long side chains or highly branched structures in all three polysaccharides, indicating that they primarily consist of linear chains.

### 2.3. Immunological Activity of Different IRPs-H Polysaccharides

#### 2.3.1. Proliferation of RAW264.7 Cells

**Figure 8:**
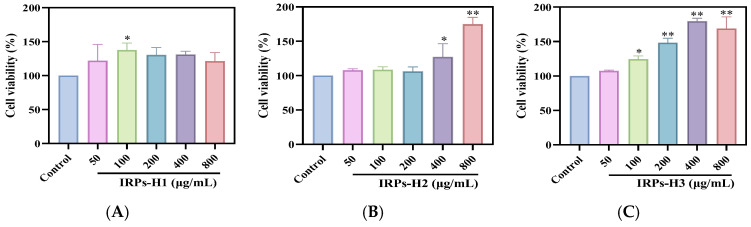
The effects of IRPs-H polysaccharides on the proliferation of RAW264.7 cells. Note: (**A**) IRPs-H1; (**B**) IRPs-H2; (**C**) IRPs-H3. * represents a significant difference compared with the blank group, *p* < 0.05; ** indicates that there was a significant difference compared with the blank group, *p* < 0.01.

Figure 8 illustrates the effects of purified IRPs-H polysaccharides on cell proliferation. Compared to the blank control, all three components significantly enhanced cell proliferation at concentrations below 800 μg/mL. Moreover, the proliferation rate increased in a dose-dependent manner. These results indicate that IRPs-H polysaccharides promote the proliferation of RAW264.7 cells and exhibit no cytotoxicity within the tested concentration range, supporting their suitability for further biological activity evaluation.

#### 2.3.2. Analysis of Phagocytosis for RAW264.7 Cells

**Figure 9:**
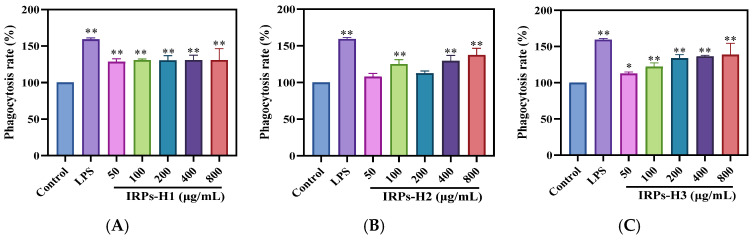
The effect of each of the IRPs-H polysaccharides on the phagocytosis of RAW264.7 cells. Note: (**A**) IRPs-H1; (**B**) IRPs-H2; (**C**) IRPs-H3. * represents a significant difference compared with the blank group, *p* < 0.05; ** indicates that there was a significant difference compared with the blank group, *p* < 0.01.

Neutral red is a pH-sensitive dye with a color transition range from pH 6.4 to 8.0, commonly used to stain cell nuclei. Its uptake by cells reflects their physiological state, as dye absorption correlates with phagocytic activity [20]. As shown in Figure 9, the neutral red uptake was significantly higher in the LPS-stimulated group compared to the blank control. Treatment with IRPs-H polysaccharides enhanced the phagocytic capacity of RAW264.7 cells in a dose-dependent manner. At higher concentrations, the phagocytic activity was comparable to that of the LPS positive control. These findings suggest that all IRPs-H components effectively enhance macrophage phagocytic function, indicating their potential immunomodulatory activity.

Dose-dependent increases in cell proliferation and phagocytic activity were observed only in IRPs-H2 and IRPs-H3, but not in IRPs-H1. This discrepancy may be attributed to differences in their molecular structure and composition. IRPs-H2 and IRPs-H3 possess more complex molecular weight distributions and higher proportions of specific monosaccharides that may enhance macrophage activation. In contrast, IRPs-H1 simpler structure and lower molecular weight components might limit its bioactivity and cellular interaction, resulting in a lack of dose-dependent effects. These structural variations likely influence their recognition by immune receptors and subsequent cellular responses.

#### 2.3.3. Morphological Changes in RAW264.7 Cells

**Figure 10:**
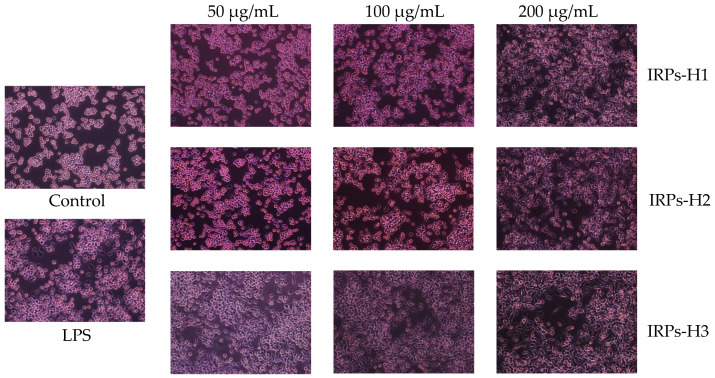
The effects of IRPs-H polysaccharides on the morphology of RAW264.7 cells.

The effects of different concentrations of IRPs-H polysaccharide components on RAW264.7 cell morphology were observed using an inverted microscope (Figure 10). Cells in the blank control group exhibited a uniform, regular round shape. In contrast, the LPS-positive control group showed marked morphological changes, including irregular shapes and pseudopodia formation. Treatment with IRPs-H components induced concentration-dependent morphological changes. With increasing concentration, cell density increased, pseudopodia became more pronounced, and the proportion of irregularly shaped cells rose. These results further confirm that IRPs-H polysaccharides promote activation and proliferation of RAW264.7 macrophages.

#### 2.3.4. Analysis of Different IRPs-H on NO Secretion in RAW264.7 Cells

**Figure 11:**
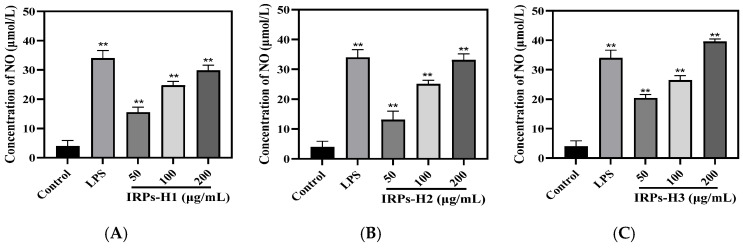
The effect of NO in RAW264.7 cells. Note: (**A**) IRPs-H1; (**B**) IRPs-H2; (**C**) IRPs-H3. ** indicates that there was a significant difference compared with the blank group, *p* < 0.01.

NO is a key bioactive molecule produced by immune cells such as macrophages. It functions as a second messenger and neurotransmitter, mediating various biological processes [21]. NO also acts as an effector molecule with potent cytotoxic activity, playing a critical role in regulating tumor cell apoptosis and inhibiting pathogenic microorganisms. Thus, NO secretion is widely used as an indicator of macrophage immune activity. As shown in Figure 11, treatment with the three IRPs-H components significantly increased NO levels in the cell culture supernatant in a dose-dependent manner. At 200 μg/mL, IRPs-H1, IRPs-H2, and IRPs-H3 induced NO release of 29.8, 33.12, and 39.56 μmol/L, respectively. Among them, IRPs-H3 showed the strongest effect, comparable to the positive control (39.99 ± 2.17 μmol/L), demonstrating a significant upregulation of NO secretion in RAW264.7 cells.

#### 2.3.5. Analysis of Different IRPs-H on Cytokines in RAW264.7 Cells

Immune macrophages play a vital role in immune responses by secreting cytokines such as TNF-α and IL-6. TNF-α is a key pro-inflammatory factor that activates immune cells, enhances inflammation, and promotes immune cell migration to sites of infection or injury [22]. It also exhibits anti-tumor effects and regulates metabolic functions. IL-6 is central to the acute-phase response, stimulating inflammatory mediator production, regulating B and T cell differentiation, supporting antibody production, and participating in tissue repair and metabolism. Both cytokines act synergistically to modulate immune responses and facilitate pathogen clearance.

**Figure 12:**
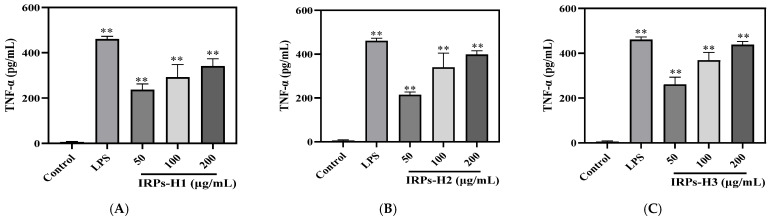

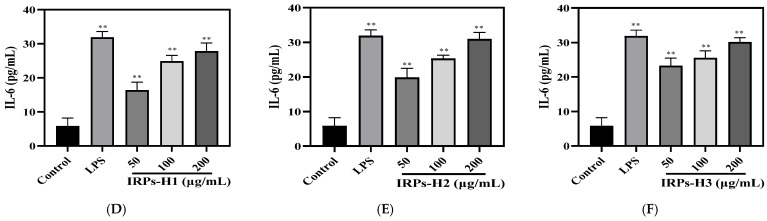
Effects of different IRPs-H polysaccharides on TNF-α secretion (**A**–**C**) and IL-6 (**D**–**F**) in RAW264.7 cells. Note: (**A**,**D**) IRPs-H1; (**B**,**E**) IRPs-H2; (**C**,**F**) IRPs-H3. ** indicates a very significant difference compared to the blank group, *p* < 0.01.

In this study, the secretion of TNF-α and IL-6 from RAW264.7 cells treated with varying concentrations of IRPs-H was measured using ELISA kits. As shown in Figure 12, cytokine levels in the blank control were within normal ranges. Treatment with IRPs-H polysaccharides significantly increased TNF-α and IL-6 secretion. Furthermore, their secretion showed a positive dose-response relationship across the tested concentrations. Notably, IRPs-H3 induced TNF-α and IL-6 release to 438.02 ± 14.14 pg/mL and 30.13 ± 1.27 pg/mL, respectively, which were comparable to those of the positive control LPS group, which had levels of 460.83 ± 16.10 pg/mL and 31.87 ± 1.72 pg/mL.

#### 2.3.6. Analysis of the Release of Reactive Oxygen Species from RAW264.7 Cells

Macrophages play a critical role in immune defense by producing ROS. As key antimicrobial agents, ROS directly damage the cellular structures of bacteria, viruses, and fungi, thereby enhancing resistance to infection. Additionally, ROS regulate immune signaling pathways, modulating inflammatory responses and cytokine production, which influence the magnitude and duration of immune activity [23]. Thus, controlled ROS release by macrophages is essential for maintaining immune defense and tissue homeostasis.

**Figure 13:**
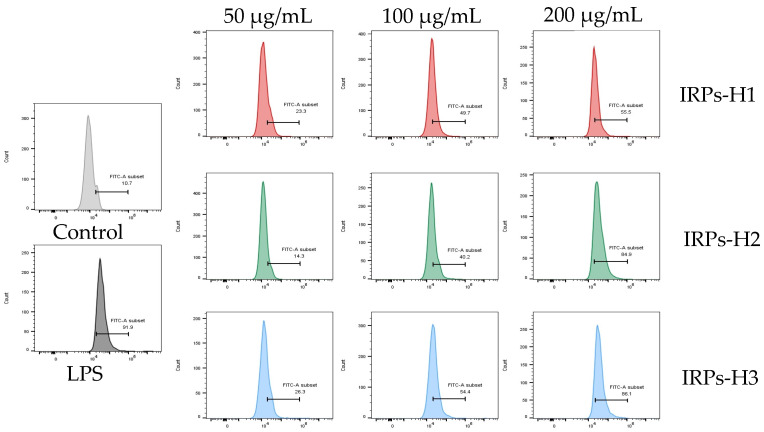
Effect of different IRPs-H on reactive oxygen species levels in RAW264.7 cells.

**Figure 14:**
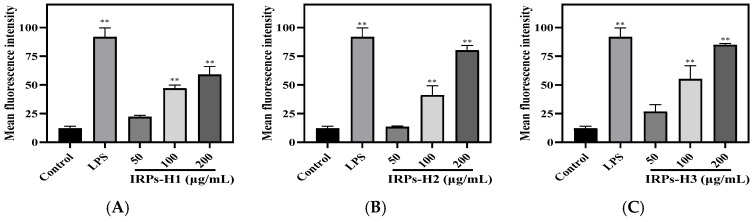
Statistics of reactive oxygen species fluorescence intensity after treatment with RAW264.7 cells with IRPs-H components. Note: (**A**) IRPs-H1; (**B**) IRPs-H2; (**C**) IRPs-H3. ** indicates that there was a significant difference compared with the blank group, *p* < 0.01.

Intracellular ROS levels were measured by flow cytometry, with results presented in Figure 13 and fluorescence intensity statistics in Figure 14. ROS levels in all treatment groups and the LPS positive control were significantly higher than in the blank control. Moreover, FITC-A fluorescence intensity increased markedly with rising treatment concentrations. Compared to the blank control (12.13 ± 1.76%), cells treated with IRPs-H polysaccharides showed significantly elevated ROS levels. IRPs-H3 induced the strongest increase (84.83 ± 1.30%, *p* < 0.01), comparable to the LPS group (91.67 ± 8.05%). These results demonstrate that IRPs-H components, at 50–200 μg/mL, effectively enhance ROS production in RAW264.7 cells, supporting their immunomodulatory activity.

## 3. Discussion

In this study, hot water extraction combined with alcohol precipitation was employed to isolate polysaccharides, demonstrating clear advantages in preserving their native structure and bioactivity. Hot water extraction is a mild method that minimizes degradation of sensitive glycosidic bonds and functional groups, thus maintaining structural integrity crucial for immunomodulatory effects [24]. Alcohol precipitation efficiently concentrates polysaccharides and removes small molecule impurities without harsh chemicals, yielding fractions suitable for bioactivity assays [25]. However, this method generally results in lower extraction efficiency compared to enzymatic, ultrasonic, or microwave-assisted techniques. Enzymatic extraction can improve yield and specificity by degrading cell wall components but introduces variability due to enzyme specificity and processing conditions [26]. Ultrasonic and microwave-assisted methods disrupt plant matrices more aggressively, enhancing yield but potentially causing polysaccharide depolymerization or conformational changes that may impair bioactivity [27]. Therefore, a trade-off exists between extraction yield and structural preservation, with hot water extraction favoring the latter in immunological studies.

Polysaccharides extracted from *Imperata cylindrica* rhizomes mainly consist of neutral sugars, predominantly glucose and xylose. Trace amounts of galacturonic acid, ribose, and glucuronic acid were also detected, indicating a heteropolysaccharide rich in hemicellulose-like components [28]. The low content of uronic acids may affect solubility and interactions with immune receptors. Compared to other Poaceae polysaccharides, *Imperata cylindrica* polysaccharides have a simpler sugar profile, with higher glucose and xylose levels but fewer acidic sugars and branching residues. These structural differences likely underlie the diverse bioactivities seen among Poaceae species. For example, arabinoxylans from *Zea mays* exhibit prebiotic effects, while β-glucan-rich fractions from *Oryza sativa* show immunomodulatory and cholesterol-lowering properties [29].

Notably, the relatively linear structure, low branching degree, and moderate molecular weight of *Imperata cylindrica* polysaccharides may facilitate receptor accessibility, such as Dectin-1 on macrophages, enhancing cellular uptake [30]. Highly branched or overly large polysaccharides often hinder receptor binding or tissue penetration. Our findings showed that IRPs-H significantly enhanced the phagocytic activity of RAW 264.7 macrophages and increased secretion of pro-inflammatory cytokines, including NO, TNF-α, and IL-6. NO, regulated by inducible nitric oxide synthase, has direct antimicrobial effects and acts as an immune signaling molecule amplifying inflammation [31]. TNF-α and IL-6 play key roles in immune cell recruitment and activation, demonstrating IRPs-H’s multifaceted regulation of innate immunity.

HPGPC analysis revealed that IRPs-H is a heterogeneous mixture with a broad molecular weight distribution (5–500 kDa). This diversity may contribute to its wide-ranging immunomodulatory effects. High molecular weight fractions (>100 kDa) generally correlate with stronger macrophage activation, while low molecular weight components (<50 kDa) may enhance bioavailability and lymphocyte stimulation [32]. And the limitation of this study lies in the insufficient analysis of the branching structure of the polysaccharides. If conditions permit, we will continue to perform methylation analysis to fully elucidate the structure of the material.

Differences in monosaccharide composition among IRPs-H fractions are crucial to their immune activity. Glucose, especially in the form of β-glucans, activates receptors like Dectin-1, enhancing macrophage phagocytosis and cytokine secretion [33]. Xylose may influence dendritic cell maturation and T-helper cell polarization, supporting adaptive immunity. Minor sugars such as rhamnose, fucose, and ribose may further modulate immune signaling via receptors like TLR4 [10]. The low uronic acid content renders these polysaccharides neutral, improving solubility and bioavailability, thus effectively stimulating both innate and adaptive immunity.

In summary, this extraction method preserves native glycosidic bonds and minimizes conformational changes, resulting in high-purity polysaccharides well-suited for immunological evaluation. We propose combining mild enzymatic pretreatment with the current extraction protocol to improve yield while maintaining structural integrity. Further comparative studies are warranted to optimize the balance between extraction efficiency and biological activity. The relatively simple yet diverse monosaccharide composition of *Imperata cylindrica* polysaccharides offers distinct immunomodulatory advantages. Their ability to activate immune cells, promote cytokine production, and modulate T cell responses underpins their significant bioactivity. Future work should focus on elucidating structure–function relationships to clarify the roles of individual components in immune regulation. Regarding the immunomodulatory and antitumor potential of IRPs-H, further optimization of extraction and purification processes is needed to improve purity, bioactivity, and stability. Additionally, detailed studies on the link between structure and immune activity, as well as preclinical safety and efficacy evaluations, will be essential for advancing their therapeutic application in immune regulation and cancer treatment.

## 4. Materials and Methods

### 4.1. Materials and Reagents

Fresh rhizome of *Imperata cylindrica* was purchased from Linyi (Shandong, China), and DEAE-52 was obtained from Shanghai Yuanye Biotechnology (Shanghai, China). Sodium chloride (AR) and ammonium hydroxide (AR) were purchased from Sinopharm Chemical Reagent Co., Ltd. (Shanghai, China). Glacial acetic acid, trifluoroacetic acid, potassium bromide (AR), iodine (AR), potassium iodide (AR), and monosaccharide standards were supplied by Shanghai Macklin Biochemical Technology Co., Ltd. (Shanghai, China). 1-Phenyl-3-methyl-5-pyrazolone was provided by Shanghai Aladdin Bio-chemical Technology Co., Ltd. (Shanghai, China). Congo red (AR) was obtained from Beijing Solarbio Science & Technology Co., Ltd. (Beijing, China). Dialysis bags (MWCO: 3000 Da) were purchased from Shanghai Yuanye Biotechnology Co., Ltd. (Shanghai, China). Dextran (AR) was acquired from the National Institutes for Food and Drug Control (Beijing, China). RAW264.7 cell line was obtained from the Cell Bank of Shanghai Institute of Life Sciences, Chinese Academy of Sciences (Shanghai, China). Fetal bovine serum was purchased from Procell Life Science & Technology Co., Ltd. (Wuhan, China). CCK-8 assay kit was supplied by Beyotime Biotechnology (Shanghai, China). Lipopolysaccharide was acquired from Sigma-Aldrich (St. Louis, MO, USA). NO detection kit was obtained from Beyotime Biotechnology (Shanghai, China). TNF-α, IL-6, and ELISA kits were provided by Quanzhou Ruixin Biotechnology Co., Ltd. (Quanzhou, China). ROS detection kit purchased from Beijing Solarbio Science & Technology Co., Ltd. (Beijing, China).

### 4.2. Isolation and Purification of IRPs-H

Fresh rhizomes of *Imperata cylindrica* were weighed and extracted using water as the solvent in a round-bottom flask. The traditional hot water extraction was performed at 100 °C for 1.5 h with a material-to-liquid ratio of 1:6 (g/mL), repeated three times [34]. The resulting IRPs-H polysaccharides were dissolved in a small volume of distilled water and subjected to high-speed centrifugation to remove precipitates. After equilibrating the chromatography column with packing material, the sample solution was gently applied along the inner wall of the column using a pipette. Sequential elution was carried out with deionized water, followed by NaCl solutions at 0.2, 0.4, and 0.6 mol/L. Fractions were collected, and their absorbance was measured by the phenol-sulfuric acid method. Finally, purified fractions were freeze-dried to obtain three distinct IRPs-H polysaccharide components.

### 4.3. Structural Elucidation of Each IRPs-H

#### 4.3.1. Ultraviolet Spectroscopy Characteristics of Each IRPs-H

The above three IRPs-H solutions were prepared as 0.5 mg/mL, and scanned by UV (2600, Shimadzu, Kyoto, Japan) in the range of 200–400 nm.

#### 4.3.2. Fourier-Transform Infrared Spectroscopy of Each IRPs-H

The completely dried KBr and the three different IRPs-H polysaccharide components were thoroughly mixed and ground using an onyx mortar. After pressing the mixture into pellets, the FT-IR (IRPrestige-21, Shimadzu, Japan) was recorded over a wavenumber range of 400 cm^−1^ to 4000 cm^−1^ [35].

#### 4.3.3. Analysis of the Monosaccharide Composition of Each IRPs-H

The monosaccharide composition of *Imperata cylindrica* polysaccharides was determined using PMP derivatization coupled with LC-MS (Agilent 1260, Triple Quad 4500; Agilent Technologies, Santa Clara, CA, USA) [36].

Exactly 10 mg of each IRPs-H powder was placed in digestion tubes, and 2 mL of 2 M trifluoroacetic acid (TFA) was added. The mixture was hydrolyzed at 110 °C for 6 h in a drying oven. After cooling to room temperature, the solution was removed, and TFA was evaporated under reduced pressure. The hydrolysate was then dissolved in 1 mL distilled water and transferred to test tubes.

Monosaccharide standards at 5 mg each were dissolved in 1 mL distilled water to prepare standard solutions. For derivatization, 100 μL of hydrolyzed IRPs-H solution or standard solution was mixed with 100 μL concentrated ammonia and 200 μL 0.5 mol/L PMP-methanol solution. The mixture was incubated at 70 °C for 30 min, then cooled to room temperature. Next, 500 μL 0.3 mol/L HCl was added to neutralize, followed by dilution with 1 mL distilled water. Chloroform (1 mL) was added for extraction, repeated three times, yielding PMP-derivatized samples.

Derivatized solutions were filtered through a 0.45 μm membrane and stored in LC vials. Separation was conducted on a Cortecs UPLC C18 column (1.6 µm, 2.1 × 100 mm) at 30 °C. Mobile phase A was acetonitrile, and B was 10 mM ammonium formate, at 0.3 mL/min flow rate. Detection wavelength was 250 nm. Mass spectrometry used multiple reaction monitoring in positive ion mode. The gradient elution program is detailed in Table 3.

#### 4.3.4. Molecular Weight Determination of Each IRPs-H

The purity and molecular weight of the polysaccharides were analyzed by HPGPC. The sample solution was filtered through a 0.22 μm membrane, and 20 μL of filtrate was injected into the system. Chromatographic separation was performed using a TSK-gel G3000 PWXL column (7 μm, 7.8 × 300 mm; TOSOH, Tokyo, Japan) at 40 °C, with 0.05 M sodium sulfate as the mobile phase at 0.5 mL/min. Detection was performed using a refractive index detector [37].

A standard curve was established with dextran standards of known molecular weights. Sample purity was evaluated by the elution profile, where a single symmetric peak indicated high purity. Molecular weight distribution was determined by comparing sample retention times with the standard curve, enabling calculation of the polysaccharides’ relative molecular weight.

#### 4.3.5. AFM Analysis of Each IRPs-H

IRPs-H1, IRPs-H2, IRPs-H3 were measured and prepared in a 0.01 mg/mL solution, which was dropped onto mica flakes, and their molecular patterns were observed by AFM (AFM5500M; Hitachi High-Tech Corporation, Tokyo, Japan) after drying [17].

#### 4.3.6. Thermal Stability Analysis of Each IRPs-H

An appropriate amount of IRPs-H1, IRPs-H, and IRPs-H3 samples were placed in a quartz crucible for real-time TGA(DTG-60; Shimadzu Corporation, Kyoto, Japan). The temperature programmed range was 30~800 °C, the heating rate was 10 °C/min, the pressure was maintained at 10 kPa, and the flow rate was 50 mL/min. The thermal stability of the sample was analyzed

#### 4.3.7. Determination of the Triple Helix Structure of Each IRPs-H

Solutions of IRPs-H1, IRPs-H2, and IRPs-H3 were prepared at 1 mg/mL, each mixed with an equal volume of 80 μmol/L Congo red solution. A 1 mol/L NaOH solution was then added dropwise to adjust the final NaOH concentrations to 0, 0.1, 0.2, 0.3, 0.4, and 0.5 mol/L. In the control group, distilled water replaced the polysaccharide solution. After standing at room temperature for 10 min, full-wavelength scans from 400 to 600 nm were recorded using a UV–visible spectrophotometer [38]. The maximum absorption wavelengths at different NaOH concentrations were analyzed to assess the triple-helix structural characteristics of IRPs-H1, IRPs-H2, and IRPs-H3.

#### 4.3.8. Branched Chain Determination of Each IRPs-H

IRPs-H1, IRPs-H2 and IRPs-H3 solutions with a concentration of 1 mg/mL were prepared. Subsequently, 2 mL solution of each polysaccharide was mixed with 1.2 mL iodine reagent (0.2% KI solution containing 0.02% I_2_). After fully mixed, the reaction mixture was scanned by ultraviolet–visible spectrophotometer at full wavelength. Then, the UV absorption characteristics in the wavelength range of 300–700 nm were analyzed.

### 4.4. Cell Culture

#### 4.4.1. Determination of the Proliferation Capacity of Each Component of IRPs-H on RAW264.7 Cells

Cell viability of each IRPs-H polysaccharide component (50–800 μg/mL) was assessed using the CCK-8 assay. Cells in the logarithmic growth phase were diluted to 5 × 10⁵ cells/mL with complete DMEM. To reduce edge effects, 200 μL of PBS was added to the outer wells of a 96-well plate. Then, 100 μL of the cell suspension was seeded into each well and incubated for 6 h to allow cell adhesion. After removing the old medium, fresh medium containing IRPs-H1, IRPs-H2, or IRPs-H3 at concentrations of 0, 50, 100, 200, 400, or 800 μg/mL was added, followed by a 24 h incubation.

After incubation, the medium was discarded, and 110 μL of CCK-8 working solution was added to each well. The plate was incubated for 1 h, and the OD_450_ was measured using a microplate reader. The relative cell viability was calculated based on the control group, which was set as 100%. This method allowed for the assessment of the effects of IRPs-H polysaccharides on cell proliferation and viability [39].

Cell viability (%) = (OD assay − OD blank)/(OD control − OD blank) × 100%(1)

The formula is as follows: among them, the control group was the cells without drug treatment, and the blank group was only supplemented with the prepared CCK-8 working solution.

#### 4.4.2. Effect of Each of the IRPs-H Components on the Phagocytic Capacity of RAW264.7 Cells

The effect of IRPs-H polysaccharides on RAW264.7 cell phagocytic activity was evaluated using neutral red staining. Log-phase cells were diluted to 5 × 10^5^ cells/mL in complete DMEM. To reduce edge effects, 200 μL PBS was added to the outer wells of a 96-well plate, followed by seeding 100 μL of the cell suspension per well. After 6 h of incubation for adhesion, the medium was replaced with fresh medium containing IRPs-H1, IRPs-H2, or IRPs-H3 at concentrations of 0, 50, 100, 200, 400, or 800 μg/mL. LPS (1 μg/mL) served as a positive control. Cells were incubated for 24 h.

After incubation, supernatants were discarded, and cells were washed 2–3 times with PBS. Then, 0.5% neutral red solution was added, and cells were incubated for 2 h. Excess dye was removed, cells washed again, and 100 μL of lysate was added. The plate was incubated for 10 min, and phagocytic activity was determined based on the amount of neutral red retained [40].

Phagocytosis = OD assay/OD blank × 100%(2)

The formula is as follows: OD assay is the absorbance value of the experimental drug administration group at absorbance 540 nm; OD blank is the absorbance value of the blank control group at absorbance 540 nm.

#### 4.4.3. The Morphological Changes in RAW264.7 Cells Were Observed by Inverted Microscope

Log-phase cells were adjusted to 5 × 10^5^ cells/mL and seeded into 6-well plates. They were divided into three groups: blank control, positive control (treated with LPS) and treatment groups (IRPs-H1, IRPs-H2, and IRPs-H3). The treatment groups were exposed to IRPs-H polysaccharides at 50, 100, and 200 μg/mL. After 24 h of incubation, cell morphology was observed and recorded using an inverted microscope (IX51; Olympus Corporation, Tokyo, Japan).

#### 4.4.4. Determination of NO Release by IRPs-H Component in RAW264.7 Cells

After photographing the cells, the supernatant was collected by centrifugation. The concentration of NO in the supernatant was measured using an NO assay kit, following the manufacturer’s instructions [41]. The effects of different IRPs-H polysaccharide components on NO secretion by RAW264.7 cells were subsequently analyzed.

#### 4.4.5. Determination of Cytokines by Each IRPs-H Component in RAW264.7 Cells

The secretion level of cytokines in the cell supernatant was determined using the TNF-α and IL-6 detection kits [39].

#### 4.4.6. Determination of the ROS from RAW264.7 Cells by Polysaccharides of Each Component of IRPs-H

According to the instructions provided with the ROS assay kit, the DCFH-DA probe working solution was prepared by diluting it to a concentration of 10 μmol/L in the base medium at a 1:1000 ratio. The working solution was then added to the cells, which were resuspended and incubated in a cell culture incubator for 20 min. During this incubation, the cells were mixed every 3–5 min to ensure complete interaction between the probe and the cells. After incubation, the cells were centrifuged and washed three times with the base medium. The cells were then resuspended in 1 mL of base medium. The fluorescence intensity of each group was measured using a flow cytometer (CytoFLEX S; Beckman Coulter, Inc., Brea, CA, USA).

### 4.5. Data Processing

All experiments were performed three times. Data were expressed as mean ± standard deviation (SD). To compare the differences between multiple groups, the data were analyzed by ANOVA. *p* < 0.05 was considered statistically significant. Origin 2021 and IBM SPSS Statistics 26 and GraphPad Prism 8 were used for statistical analysis.

## 5. Conclusions

This study systematically evaluated the immunomodulatory effects of purified polysaccharide components from the rhizome of *Imperata cylindrica* on RAW264.7 cells. The CCK-8 assay showed that these polysaccharides exhibited no cytotoxicity at 50–800 μg/mL and promoted cell proliferation. Further functional assays demonstrated that all three polysaccharide components significantly enhanced the phagocytic activity of RAW264.7 cells, suggesting their role in boosting macrophage-mediated immunity. Morphological analysis via inverted microscopy revealed increased cell size and pseudopodia formation, characteristic of macrophage activation. Quantitative measurement of cell supernatants confirmed a dose-dependent increase in the secretion of immune mediators, including NO, IL-6, and TNF-α, supporting their immunomodulatory potential. Flow cytometry further showed that, at 50–200 μg/mL, all three IRPs-H components significantly increased ROS production, indicating activation at the molecular level.

In conclusion, the three IRPs-H polysaccharide components exhibit robust immunomodulatory activity through multiple mechanisms: promoting proliferation, enhancing phagocytosis, regulating immune molecule secretion, and inducing ROS production. These results highlight the potential of the rhizomes of *Imperata cylindrica* polysaccharides as effective immune modulators.

## Figures and Tables

**Table 1:** Monosaccharide composition table of different IRPs-H polysaccharides.

	Man	Rha	GlcA	GalA	Glc	Gal	Xyl	Rib	Fuc
IRPs-H1 (%)	3.18	1.42	-	1.47	46.43	21.29	25.41	-	0.57
IRPs-H2 (%)	1.61	3.74	-	-	34.75	5.8	42.88	6.54	4.53
IRPs-H3 (%)	0.47	16.85	3.11	-	48.01	4.05	17.04	2.33	8.13

**Table 2:** Molecular weight distribution table of different IRPs-H polysaccharides.

Polysaccharides	Peak	Time (min)	Mw (kDa)
IRPs-H1	1	14.094	6.9
	2	17.231	0.3
IRPs-H2	1	11.742	66.55
	2	14.114	6.8
	3	17.233	0.3
IRPs-H3	1	10.542	210.7
	2	13.749	9.2
	3	14.87	3.3
	4	17.239	0.3

**Table 3:** Mobile phase gradient elution program.

Time (min)	A%	B%	Peak
0	13	87	1
0.5	13	87	2
2	16	84	1
15	21	79	2
16	60	40	3
17	90	10	1
17.1	13	87	2
20	13	87	3

## Data Availability

Data will be made available on request.

## References

[1] Zeng P., Li J., Chen Y., Zhang L. (2019). The Structures and Biological Functions of Polysaccharides from Traditional Chinese Herbs. Prog. Mol. Biol. Transl. Sci..

[2] Zhao T., Yang M., Ma L., Liu X., Ding Q., Chai G., Lu Y., Wei H., Zhang S., Ding C. (2023). Structural Modification and Biological Activity of Polysaccharides. Molecules.

[3] Schepetkin I.A., Quinn M.T. (2006). Botanical Polysaccharides: Macrophage Immunomodulation and Therapeutic Potential. Int. Immunopharmacol..

[4] Zhou L., Liu Z., Wang Z., Yu S., Long T., Zhou X., Bao Y. (2017). Astragalus Polysaccharides Exerts Immunomodulatory Effects via TLR4-Mediated MyD88-Dependent Signaling Pathway in Vitro and in Vivo. Sci. Rep..

[5] Wu J., Yu G., Zhang X., Staiger M.P., Gupta T.B., Yao H., Wu X. (2024). A Fructan-Type Garlic Polysaccharide Upregulates Immune Responses in Macrophage Cells and in Immunosuppressive Mice. Carbohydr. Polym..

[6] Sato W., Takeshita K., Tsuboi M., Kanamori M., Ishibashi K., Miura N.N., Adachi Y., Ohno N. (2015). Specificity of the Immunomodulating Activity of Sasa Veitchii (Japanese Folk Medicine Kumazasa) to Fungal Polysaccharides. Int. J. Med. Mushrooms.

[7] Shin M.-R., Lee J.H., Lee J.A., Kim M.J., Park H.-J., Park B.W., Seo S.B., Roh S.-S. (2021). Immunomodulatory and Anti-Inflammatory Effects of Phellinus Linteus Mycelium. BMC Complement. Med. Ther..

[8] Jung Y.-K., Shin D. (2021). *Imperata cylindrica*: A Review of Phytochemistry, Pharmacology, and Industrial Applications. Molecules.

[9] Fu L.-N., Chen L.-Y., Liu R.-H., Chen D.-F. (2010). [Chemical Constituents of Rhizoma Imperatae and Their Anti-Complementary Activity]. Zhong Yao Cai = Zhongyaocai = J. Chin. Med. Mater..

[10] Zhou C., Yin S., Yu Z., Feng Y., Wei K., Ma W., Ge L., Yan Z., Zhu R. (2018). Preliminary Characterization, Antioxidant and Hepatoprotective Activities of Polysaccharides from Taishan Pinus Massoniana Pollen. Molecules.

[11] Mi S., Yan W., Wei L., Xiong X., Tian Y., Lu Q., Mu L. (2025). Structural Characterization and Hypoglycemic Activity of a Polysaccharide from Imperatae Rhizoma. Int. J. Biol. Macromol..

[12] Chen L., Chen Z., Wang C., Luo Y., Luo Y., Duan M., Liu R. (2015). [Protective Effects of Different Extracts of Imperatae Rhizoma in Rats with Adriamycin Nephrosis and Influence on Expression of TGF-Β1, and NF-κB P65]. Zhong Yao Cai = Zhongyaocai = J. Chin. Med. Mater..

[13] Jee W., Ko H.M., Park D.-I., Park Y.-R., Park S.-M., Kim H., Na Y.-C., Jung J.H., Jang H.-J. (2023). Momordicae Semen Inhibits Migration and Induces Apoptotic Cell Death by Regulating C-Myc and CNOT2 in Human Pancreatic Cancer Cells. Sci. Rep..

[14] Zhao Z.-H., Ju X.-Y., Wang K.-W., Chen X.-J., Sun H.-X., Cheng K.-J. (2022). Structure Characterization, Antioxidant and Immunomodulatory Activities of Polysaccharide from *Pteridium aquilinum* (L.) Kuhn. Foods.

[15] Shen Q., He Z., Ding Y., Sun L. (2023). Effect of Different Drying Methods on the Quality and Nonvolatile Flavor Components of Oudemansiella Raphanipes. Foods.

[16] Wang Z., Zheng Y., Lai Z., Hu X., Wang L., Wang X., Li Z., Gao M., Yang Y., Wang Q. (2024). Effect of Monosaccharide Composition and Proportion on the Bioactivity of Polysaccharides: A Review. Int. J. Biol. Macromol..

[17] Zhong W., Yang C., Zhang Y., Liu Y., Yang D. (2022). The Chemical Profiling and Anticancer Potential of Functional Polysaccharides from Flos Sophorae Immaturus. Molecules.

[18] Yu W., Xiong Y., Liu M., Zeng D., Zhao H., Liu J., Lu W. (2023). Structural Analysis and Attenuates Hyperuricemic Nephropathy of Dextran from the *Imperata cylindrica* Beauv. Var. Major (Nees) C. E. Hubb. Carbohydr. Polym..

[19] Liu Y., Ran L., Wang Y., Wan P., Zhou H. (2023). Basic Characterization, Antioxidant and Immunomodulatory Activities of Polysaccharides from Sea Buckthorn Leaves. Fitoterapia.

[20] Fernandez M.A., Marette A. (2017). Potential Health Benefits of Combining Yogurt and Fruits Based on Their Probiotic and Prebiotic Properties. Adv. Nutr..

[21] Chen J., Tian S., Shu X., Du H., Li N., Wang J. (2016). Extraction, Characterization and Immunological Activity of Polysaccharides from Rhizoma Gastrodiae. Int. J. Mol. Sci..

[22] Li L., Li H., Qian J., He Y., Zheng J., Lu Z., Xu Z., Shi J. (2016). Structural and Immunological Activity Characterization of a Polysaccharide Isolated from Meretrix Meretrix Linnaeus. Mar. Drugs.

[23] Shen Y., Zhao H., Wang X., Wu S., Wang Y., Wang C., Zhang Y., Zhao H. (2024). Unraveling the Web of Defense: The Crucial Role of Polysaccharides in Immunity. Front. Immunol..

[24] Sha A., Li Y. (2025). Preparation, Structural Characterization, Bioactivities, and Potential Clinical Applications of the Polysaccharides from Paris Polyphylla: A Review. Front. Pharmacol..

[25] Li J.H., Zhu Y.Y., Gu F.T., Wu J.Y. (2023). Efficient Isolation of Immunostimulatory Polysaccharides from Lentinula Edodes by Autoclaving-Ultrasonication Extraction and Fractional Precipitation. Int. J. Biol. Macromol..

[26] Wang L., Yang Y., Tan H.-Y., Li S., Feng Y. (2020). Protective Actions of Acidic Hydrolysates of Polysaccharide Extracted from Mactra Veneriformis against Chemical-Induced Acute Liver Damage. Front. Pharmacol..

[27] Wang Z., Zhou X., Sheng L., Zhang D., Zheng X., Pan Y., Yu X., Liang X., Wang Q., Wang B. (2023). Effect of Ultrasonic Degradation on the Structural Feature, Physicochemical Property and Bioactivity of Plant and Microbial Polysaccharides: A Review. Int. J. Biol. Macromol..

[28] Yin F., Lin P., Yu W.-Q., Shen N., Li Y., Guo S.-D. (2021). The Cordyceps Militaris-Derived Polysaccharide CM1 Alleviates Atherosclerosis in LDLR(-/-) Mice by Improving Hyperlipidemia. Front. Mol. Biosci..

[29] Kwansang J., Chen C.-J., Chaiprateep E.-O. (2022). Optimization of Water-Based Ultrasonic-Microwave Assisted Extraction (UMAE) of Bioactive Compounds from Garcinia Mangostana Pericarp. J. Complement. Integr. Med..

[30] Chen Z., Wang C., Su J., Liang G., Tan S., Bi Y., Kong F., Wang Z. (2024). Extraction of Pithecellobium Clypearia Benth Polysaccharides by Dual-Frequency Ultrasound-Assisted Extraction: Structural Characterization, Antioxidant, Hypoglycemic and Anti-Hyperlipidemic Activities. Ultrason. Sonochem..

[31] Li W., Zhou Q., Lv B., Li N., Bian X., Chen L., Kong M., Shen Y., Zheng W., Zhang J. (2024). Ganoderma Lucidum Polysaccharide Supplementation Significantly Activates T-Cell-Mediated Antitumor Immunity and Enhances Anti-PD-1 Immunotherapy Efficacy in Colorectal Cancer. J. Agric. Food Chem..

[32] Lin P., Chen L., Huang X., Xiao F., Fu L., Jing D., Wang J., Zhang H., Sun L., Wu Y. (2022). Structural Characteristics of Polysaccharide GP2a in Gardenia Jasminoides and Its Immunomodulatory Effect on Macrophages. Int. J. Mol. Sci..

[33] Ma Y., Wang Z., Arifeen M.Z.U., Xue Y., Yuan S., Liu C. (2022). Structure and Bioactivity of Polysaccharide from a Subseafloor Strain of Schizophyllum Commune 20R-7-F01. Int. J. Biol. Macromol..

[34] Lu Y., Jia Y., Xue Z., Li N., Liu J., Chen H. (2021). Recent Developments in Inonotus Obliquus (*Chaga mushroom*) Polysaccharides: Isolation, Structural Characteristics, Biological Activities and Application. Polymers.

[35] Liang L., Qiu H., Liu Y., Liu Y., Weng L., Zhong W., Meng F. (2023). Exploring the Potential of Ume-Derived Proanthocyanidins: Novel Applications for Blueberry Preservation. Front. Microbiol..

[36] Wang L., Lian J., Zheng Q., Wang L., Wang Y., Yang D. (2022). Composition Analysis and Prebiotics Properties of Polysaccharides Extracted from Lepista Sordida Submerged Cultivation Mycelium. Front. Microbiol..

[37] Zhou S., Huang G. (2021). Preparation, Structure and Activity of Polysaccharide Phosphate Esters. Biomed. Pharmacother..

[38] Qiu Y., Batool Z., Liu R., Sui G., Sheng B., Zheng X., Xu D. (2020). Characterization and immunological activity of polysaccharides from *Potentilla chinensis*. Int. J. Biol. Macromol..

[39] Gu J., Zhang H., Wen C., Zhang J., He Y., Ma H., Duan Y. (2020). Purification, Characterization, Antioxidant and Immunological Activity of Polysaccharide from *Sagittaria sagittifolia* L. Food Res. Int..

[40] Hao W., Wang S., Zhao J., Li S. (2020). Effects of Extraction Methods on Immunology Activity and Chemical Profiles of *Lycium barbarum* Polysaccharides. J. Pharm. Biomed. Anal..

[41] Li C., Zhang Y., Zhao C., Fu X. (2023). Physicochemical Characterization, Antioxidative and Immunoregulatory Activity of Polysaccharides from the Flower of *Hylocereus undatus* (Haw.) Britton et Rose. Int. J. Biol. Macromol..

